# Relative validity of a web-based food frequency questionnaire for Danish adolescents

**DOI:** 10.1186/s12937-018-0312-7

**Published:** 2018-01-12

**Authors:** Anne A. Bjerregaard, Thorhallur I. Halldorsson, Freja B. Kampmann, Sjurdur F. Olsen, Inge Tetens

**Affiliations:** 1 Center for Fetal Programming, Department of Epidemiology Research, Statens Serum Institut, Artillerivej 5, 2300 Copenhagen, Denmark; 2 The Unit for Nutrition Research, Faculty of Food Science and Nutrition, School of Health Sciences, University of Iceland, Sæmundargata 2, 101 Reykjavík, Iceland; 3 Division for Diet, Disease Prevention and Toxicology, National Food Institute, Technical University of Denmark, DTU Food, Kemitorvet, building, 202 Kgs. Lyngby, Denmark; 4 Department of Endocrinology, Diabetes and Metabolism, Rigshospitalet, Copenhagen, Denmark; 5 The Danish Diabetes Academy, Odense, Denmark; 6 Vitality - Centre for Good Older Lives, Department of Nutrition, Exercise, and Sports, University of Copenhagen, University of Copenhagen, Rolighedsvej 26, 1958 Frederiksberg C, Denmark

**Keywords:** Dietary assessment, School-age children, Diet, Dietary intake, Cohort study, Food groups, Nutrients, Diet recall

## Abstract

**Background:**

With increased focus on dietary intake among youth and risk of diseases later in life, it is of importance, prior to assessing diet-disease relationships, to examine the validity of the dietary assessment tool. This study’s objective was to evaluate the relative validity of a self-administered web-based FFQ among Danish children aged 12 to 15 years.

**Methods:**

From a nested sub-cohort within the Danish National Birth Cohort, 124 adolescents participated. Four weeks after completion of the FFQ, adolescents were invited to complete three telephone-based 24HRs; administered 4 weeks apart. Mean or median intakes of nutrients and food groups estimated from the FFQ were compared with the mean of 3x24HRs. To assess the level of ranking we calculated the proportion of correctly classified into the same quartile, and the proportion of misclassified (into the opposite quartile). Spearman’s correlation coefficients and de-attenuated coefficients were calculated to assess agreement between the FFQ and 24HRs.

**Results:**

The mean percentage of all food groups, for adolescents classified into the same and opposite quartile was 35 and 7.5%, respectively. Mean Spearman’s correlation was 0.28 for food groups and 0.35 for nutrients, respectively. Adjustment for energy and within-person variation in the 24HRs had little effect on the magnitude of the correlations for food groups and nutrients. We found overestimation by the FFQ compared with the 24HRs for fish, fruits, vegetables, oils and dressing and underestimation by the FFQ for meat/poultry and sweets. Median intake of beverages, dairy, bread, cereals, the mean total energy and carbohydrate intake did not differ significantly between the two methods.

**Conclusion:**

The relative validity of the FFQ compared with the 3x24HRs showed that the ranking ability differed across food groups and nutrients with best ranking for estimated intake of dairy, fruits, and oils and dressing. Larger variation was observed for fish, sweets and vegetables. For nutrients, the ranking ability was acceptable for fatty acids and iron. When evaluating estimates from the FFQ among Danish adolescents these findings should be considered.

**Electronic supplementary material:**

The online version of this article (10.1186/s12937-018-0312-7) contains supplementary material, which is available to authorized users.

## Background

Dietary intake among adolescents has consistently been associated with later health and disease risk in adult life [1, 2]. Diet-disease associations measured in adolescence are often based on dietary intake estimated from a food frequency questionnaire (FFQ) particularly in largescale studies [2–6]. The FFQ method is often self-administered which is more cost-effective, less intrusive, and less time-consuming for the participants compared with other dietary assessment methods such as multiple 24-h recalls (24HR) and food records [7]. However, prior to assessing diet-disease relationships, the validity of the dietary assessment tool must be examined [8].

Validity refers to the degree to which an assessment method captures true dietary intake [9]. Such assessment requires comparison with ‘a gold standard’ method which, in reality, is rarely available. Therefore researchers are often left with the option of examining relative validity by comparing one dietary assessment method with another method that has a different error structure [10].

Although a 24HR relies on memory, as does the FFQ, 24HRs are common in relative validity studies using FFQs among adolescents [11, 12]. FFQs are in general considered suitable or valid for ranking adolescents at group level with correlation coefficients ranging from 0.2 to 0.8 [11–14]. In terms of what can be considered as acceptable validity in such studies, Cade et al. suggested a cut-off of 0.40 as an acceptable correlation [15]. However, such an approach has been criticised by others, as a correlation of 0.50 indicates that 75% of the variance in estimates is error, thus increasing the risk of misclassification in association studies [16]. Nevertheless, correlation coefficients are still the main statistical approach in validation studies which ensures the possibility for some comparison across studies. A recent meta-analysis found correlation coefficients above 0.40 for most nutrients in 16 validity studies comparing an FFQ to food record or 24HRs among 13- to 17-year-old adolescents [10]. However, high heterogeneity was also reported in validation studies with administration mode and recall interval as strongest contributors [10]. Other factors which potentially influence validity are the respondent’s memory and ability to identify and quantify consumed foods [17, 18]. In addition, compared with validation studies in adults, a higher degree of intrusion and omission among children has been demonstrated [19]. Thus, it is important to evaluate if an FFQ can assess dietary intake and rank adolescents in terms of energy, nutrient, and food intakes. To our knowledge, a web-based FFQ has not previously been validated among Danish adolescents. The aim of this study therefore was to evaluate the relative validity of a web-based FFQ using a nested sub-cohort of adolescents aged 12 to 15 years old, within the Danish National Birth Cohort (DNBC).

## Methods

### Subjects

The DNBC includes information from 101,042 pregnancies and is described in details elsewhere [20]. In brief, information on participants was recorded through telephone interviews in gestation weeks (GW) 12 and 30, and 6 and 18 months postpartum. Maternal diet was assessed in GW 25 using an extensive FFQ [20]. Additional available offspring data were collected at age 7 and 11.

Data for the present study were obtained in a nested sub-cohort within the DNBC called the Diabetes and Women’s Health Study, which ran from May 2012 to April 2014 [21]. In a group of women who had had gestational diabetes (GDM) and a group of women who had had a normal pregnancy, insulin sensitivity, risk of type 2 diabetes, and risk of diabetes in offspring aged 9–15 years were investigated [21]. This sub-cohort provided an opportunity to invite adolescents between 12 to 15 years of age to participate in the validation study. The invited adolescents were offspring of women already in the DNBC cohort. These adolescents were all considered relatively healthy in terms of non-communicable diseases, and no exclusion criteria were applied.

### Design

All offspring of mothers participating in the Diabetes and Women’s Health Study were invited to fill out a web-based FFQ during their pre-planned clinical visit. After the clinic visit, 178 adolescents were asked to participate in the 24HRs by e-mail (45), phone (12) or hand-out at the clinic (121). Follow-up on the invitation was done by e-mail and by phone call approximately 7 days after the invitation was received and 7 days apart. The first 24HR was carried out 4 weeks after the FFQ followed by two recalls 4 weeks apart. Three non-consecutive telephone-based 24HRs were conducted by four trained nutritionists, and each interview took approximately 25 min. In order to make the 24HRs represent weekdays and weekends, a set of three different days were randomly designed. A set of 3 days was distributed to the participants, ensuring equal distribution of all 7 days of the week at group level. Participants were not informed beforehand as to which day the 24HR would be performed. They only received a mobile text message on the day the recall took place, to arrange a suitable time for the conversation. All participants automatically entered into a competition to win one of three Ipads.

### The food frequency questionnaire

The web-based self-administered FFQ developed for this study was based on the validated youth/adolescent questionnaire (YAQ) from the Growing Up Today Study [13, 22] which is a follow-up study of children born to women in the Nurses’ Health Study 2 (NHS-2). The YAQ was translated into Danish and modified to include typical Danish foods based on the reports National Danish Dietary Habits and Physical activity [23, 24]. The aim of modifying the YAQ was to make sure that the FFQ reflects Danish dietary patterns and thus intake of Danish foods and beverages. We previously evaluated the reproducibility of the FFQ and showed high concordance between two repeated measurements with the FFQ with a Spearman’s correlation coefficient for energy of 0.78 [25].

The FFQ included 145 frequency questions on food items clustered into 8 food groups (number of food items in brackets): beverages (18), dairy (8), bread and cereals including butter on bread (14), spread on bread (14), cold and warm dishes (25), side dishes and condiments (18), fruits and vegetables (29), snacks and desserts (20). Frequency scales ranged from “did not drink/consume the last month” to “2 times or more per day” or “4 times or more per day” and the recall period was 1 month. Portion sizes were predefined for dairy products (bowl), breakfast cereals (bowl), and beverages (glass/bottle), slides of bread, fruits (pc.), selected vegetables (pc.), and cake (pc.), whereas no portion size was given for the remaining items. Portion sizes were based on standard portions developed by the National Food Institute in Denmark [26]. Additional information on food allergy, other foods the participant may wish to avoid and any major changes in food habits during the last month was included in three open-end questions.

Before the FFQ, questions on age, gender, and self-reported height and weight were listed. Following the FFQ, questions on (number of questions in parenthesis); meal habits (15), physical activity (11) and puberty (6) were listed. Finally, the participants were asked to which degree they completed the questionnaire themselves or received help from an adult. Written instruction on how to complete the questionnaire was given at the beginning of the questionnaire together with short examples of answers. Time for filling in the FFQ was approximately 40 min. The FFQ was based on the online html program Limesurvey.

### The 24-h recalls

To conduct the 24HRs, a structured interview guide was developed, with questions in a chronological order covering daily meal patterns including promt questions. Dietary intake reported by the participant was reported directly into a pre-coded Danish food record by the interviewer. The pre-coded Danish food record is a semi-closed questionnaire with answer categories for the most commonly consumed dishes according to meal patterns in Denmark as used in the nationally representative survey of Dietary Habits and Physical Activity (DANSDA) in 2011–2013 [27]. Open answers were available in each section if consumed foods were not found in the pre-coded categories. Household measures were used for quantification of portion sizes. After the interview, answers were reviewed orally together with the adolescent to make sure everything he/she had consumed was recorded.

### Calculation foods and nutrient intakes from the FFQ and the 24HRs

Frequencies of intake were computed into grams per day using nutrition software Foodcalc v.3 [28]. Nutritional calculations for the FFQ were done based on assumptions of standard portion sizes. The 24HRs were entered into an online registration system (fa.kostvaner.dk, template by cmsimple-styles.com) developed at the Danish National Food Institute (Tue Christensen, personal communication). National food composition tables [29] were used for both the FFQ and the 24HRs.

### Statistics

All statistical analyses were performed in the statistical program SAS version 9.4 (SAS Institute, Cary, NC, USA). Participant characteristics were evaluated using descriptive statistics. Age- and gender-specific cut-offs for overweight and obesity among the offspring were based on values provided by the International Obesity Task Force (IOTF) [30]. Parental educational level was retrieved from the maternal FFQ in the DNBC.

We presented median along with 25^th^ and 75^th^ percentile for food groups and nutrients since data were non-normally distributed both before and after log-transformation (except energy percent of macronutrients which were normally distributed). Paired t-test was used to compare differences between the two methods for normally distributed variables while Wilcoxon rank test was used for skewed variables. To compare differences in estimated intake between the two methods the median difference in percentages was calculated according to the formula: ((FFQ - 24HR)/24HR)*100. Misclassification analysis (into quantiles) including weighted Kappa was applied in order to test whether the FFQ ranked adolescents according to magnitude of dietary and nutrient intake by comparison to the mean of the three 24HRs. It has been suggested that at least 50% of subjects should be classified into the same category, no more than 10% should be classified into the opposite category, and Cohen’s weighted Kappa should preferably be above 0.4 [31]. Bland-Altman plots were used to elaborate whether the differences between the two methods were constant across the range of measurements, and mean intake was plotted against mean difference [32]. The association between food and nutrient intakes estimated with the two methods was assessed using crude, energy adjusted and de-attenuated Spearman’s correlation coefficients. Correlation coefficients were adjusted using the ratio of within- and between-person variation assessed on the basis of the three 24HRs. These de-attenuated correlation coefficients should provide an estimate similar to that obtained with a higher number of 24HRs [9]. Adjustment for energy was performed with the residual method [9].

To estimate the degree of selection as a result of our recruitment strategy we compared characteristics of participants with those who did not wish to participate in the validation study (non-participants). Differences in characteristics of participants born to GDMmothers vs. other offspring participants were also compared. Finally, characteristics of our participants were compared with those in the full source population, i.e. the DNBC 14-year follow-up study. For these comparisons we applied analysis of variance.

## Results

The mean age (SD) and body mass index (BMI) for the 124 participants were 13.2 (0.7) years and 19.1 kg/m^2^, respectively (Table 1). Among participants, 62% were born to GDMmothers, and 27.4% were overweight or obese (Table 1). The relative validity of the FFQ was evaluated by comparing estimated intake of food groups, energy and nutrients with the average of three 24HRs (Table 2). We found significant difference between the FFQ and the mean of three 24HRs for the majority of food groups. The food groups; fish, fruits, vegetables, and oils and dressing were overestimated by the FFQ when compared with the mean of three 24HRs, whereas meat/poultry and sweets were underestimated by the FFQ. We found no significant difference for beverages, dairy, bread, and cereals. Median difference (%) revealed the smallest differences for beverages (+10%), bread (−10%), and dairy (−5%) and larger differences for fish (+1000%), fruits (+121%), and sweets (−70%) (Table 2).

**Table 1:** Participant characteristics (*n* = 124, 52% girls)

	All	Girls	Boys
Age, years (mean, SD)	13.2 (0.7)	13.3 (0.6)	13.2 (0.7)
Height, cm (mean, SD)	165.9 (8.3)	164.7 (7.9)	167.4 (8.5)
BMI^a^ (kg/m^2^) (mean, SD)	19.1 (7.9)	19.2 (7.9)	19.1 (8.3)
Overweight^a^ (%)	13.7	15	12
Obese^a^ (%)	13.7	12	15
Born to GDM mothers^b^ (%)	62	57	68
Parental educational level (%)			
High level of education	21	26	15
Medium level of education	32.5	29	36
Skilled workers	32.5	30	36
Student	0.8	1.5	0
Unskilled	12	14	11
Unemployed	0.8	0	1.8

*BMI* body mass index = weight (kg)/height (m)^2^ *GDM* gestational diabetes mellitus

^a^Classification by Cole et al. [30]

^b^Adolescents born to mothers with registered gestational diabetes

**Table 2:** Relative validity: mean and median intake of food groups, energy and nutrients from FFQ and 3x24HR, percentage difference, and percentage agreement in categories (*n* = 124)

	FFQ		3x24HR		Median diff. %	Classification (%)		
Food groups (g/d)	Median	q1-q3	Median	q1-q3		Same quartile	Opposite quartile	Weighted Kappa
Beverages	1131	648–1273	1032	821–1464	−10	35	9	0.20
Dairy	319	182–636	335	182–521	−4.7	46	6	0.43
Bread	185	109–275	204	146–258	−9	28	7	0.12
Cereals	27	14–50	24	9–52	12	35	6	0.25
Meats/poultry	82^*^	56–118	107	69–139	−23	23	7	0.05
Fish	11^*^	6–18	1	0–16	1000	32	18	n.a.
Fruits	126^**^	52–247	57	9–135	121	43	6	0.32
Vegetables	116^*^	65–171	91	62–130	27	32	9	0.12
Sweets	17^**^	10–28	57	24–108	−70	33	4	0.20
Oils & dressing	36^*^	22–47	26	18–51	38	38	3	0.32
Nutrients	Mean or Median	SD or q1-q3	Mean or Median	SD or q1-q3				
Energy (MJ/d)	7.6	5.7–9.7	8.5	7.0–11.0	−10	36	5	0.26
Protein (E%)	15^**^	2.7	13	2.5	15	37	6	0.24
Fat (E%)	34^*^	6.3	35	6.0	−3	34	8	0.24
SFA (g/d)	26^**^	19–33	33	23–44	−21	40	2	0.34
MUFA (g/d)	24^*^	16–31	27	20–36	−11	45	2	0.39
PUFA (g/d)	12	8–15	11	9–15	9	36	3	0.29
Carbohydrate (E%)	51	6.5	51	6.0	0	25	14	0.10
Added sugar (g/d)	26^**^	15–38	41	25–69	−36	31	5	0.23
Dietary fiber (g/d)	21	15–32	18	15–24	16	41	5	0.30
Vitamin C (mg/d)	66^**^	43–100	49	33–69	34	31	7	0.14
Calcium (mg/d)	867	647–1374	873	672–1229	−0.7	39	6	0.33
Iron (mg/d)	8.3	6.3–12.5	8.3	6.7–10.8	0	40	4	0.29

Variables that reached normality after log-transformation were beverages, dairy, bread, oils & dressing, energy, SFA, MUFA, PUFA, added sugar, dietary fibers, vitamin C, calcium and iron whereas cereals, meat/poultry, fish, fruit, vegetables and sweets did not reach normality after log-transformation

Median difference in percent = median (FFQ-24HR/24HR)*100

*FFQ* food frequency questionnaire, *24HR*, 24-h recalls, *SFA* saturated fatty acids, *MUFA* monounsaturated fatty acids, *PUFA* polyunsaturated fatty acids

*p < 0.05 significantly different from 24HR

**p < 0.001 significantly different from 24HR

For nutrients, there were no significant differences in total energy, polyunsaturated fatty acids (PUFA), energy percent (E%) from carbohydrate, dietary fibres, calcium and iron. Protein E% and vitamin C were overestimated, while the remaining nutrients were underestimated by the FFQ in comparison with the three 24HRs. The median differences (%) showed smallest differences (or null for carbohydrate E% and iron) for fat E% (−3%) and calcium (−0.7), whereas the largest degree of under- or overestimation was seen for added sugar (−36%) and vitamin C (+34%) (Table 2).

**Fig. 1:**
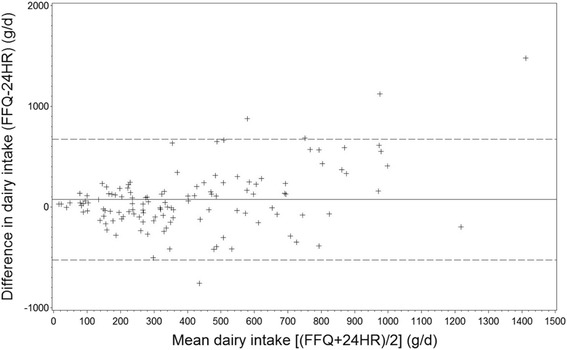
Bland-Altman plot for dairy intake for the two methods FFQ and mean of three 24HR among participants (*n* = 124). The difference in intake is plottet v. the mean intake from the two methods. ───── represents the mean difference ----- represents the 95% limits of agreement

**Fig. 2:**
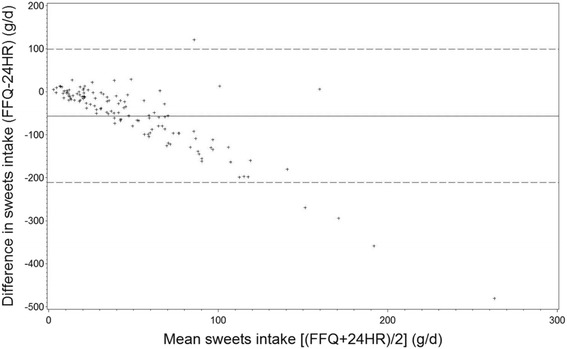
Bland-Altman plot for sweets intake for the two methods FFQ and mean of three 24HR among participants (*n* = 124). The difference in intake is plottet v. the mean intake from the two methods. ───── represents the mean difference ----- represents the 95% limits of agreement

Consistency between the two methods was also examined using Bland-Altman plots. These plots for beverages, dairy, meat/poultry, vitamin C, and calcium revealed some outliers, but no systematic patterns were observed (exemplified with dairy Fig. 1). For the remaining variables, the plots indicated a tendency towards higher difference between the FFQ and the 24HRs at higher mean intakes. This indicates a higher degree of overestimation and underestimation at higher intakes (exemplified with sweets Fig. 2). The mean difference in intake of e.g. sweets was below zero, confirming an underestimation by the FFQ. The pattern of observations indicated a higher degree of underestimation at higher mean intakes.

Misclassification (into the opposite quartile) was below 10% for all food groups and nutrients, except for fish and carbohydrate E% (Table 2). Correct classification ranged from 23% (meat/poultry) to 46% (dairy), while nutrients ranged from 25% (carbohydrate E%) to 45% (monounsaturated fatty acids (MUFA)). Weighted Kappa values ranged from 0.05 (meats/poultry) to 0.43 (dairy) (Table 2).

Spearman’s correlation coefficients for food groups ranged from 0.03 (fish) to 0.60 (dairy). Three of the 10 food groups and 5 of the 12 nutrients examined had Spearman’s correlations above 0.4. These were dairy, fruits, oils and dressing, saturated fatty acids (SFA), MUFA, PUFA, calcium, and iron (Table 3). For energy, the Spearman’s correlation coefficient was 0.38 and the mean correlation coefficient for nutrients was 0.35 (Table 3). De-attenuation improved Spearman’s correlation coefficients lightly for most variables, while further adjustment for energy only improved the correlation coefficients for MUFA (from 0.54 to 0.60) (Table 3).

**Table 3:** Spearman correlation coefficient between FFQ and 3x24HR (*n* = 124)

	Spearman correlation coefficients *r*		
Food groups (g/d)	crude	De-attenuated	Energy adjusted & de-attenuated
Beverages	0.22^*^	0.24^*^	0.24^*^
Dairy	0.60^**^	0.69^**^	0.61^**^
Bread	0.19^*^	0.19^*^	0.19^*^
Cereals	0.33^*^	0.35^*^	0.35^*^
Meats/poultry	0.16	0.17	0.16
Fish	0.03	0.03	0.03
Fruits	0.40^**^	0.43^**^	0.41^**^
Vegetables	0.13	0.13	0.13
Sweets	0.31^*^	0.32^*^	0.31^*^
Oils & dressing	0.46^**^	0.48^**^	0.47^**^
Nutrients			
Energy (MJ/d)	0.38^**^	0.54^**^	
Protein (E%)	0.28	0.32	
Fat (E%)	0.27^*^	0.28^*^	
SFA (g/d)	0.51^**^	0.54^**^	0.53^**^
MUFA (g/d)	0.52^**^	0.54^**^	0.60^**^
PUFA (g/d)	0.47^**^	0.48^**^	0.47^**^
Carbohydrate (E%)	0.09	0.09	
Added sugar (g/d)	0.37^**^	0.38^**^	0.39^**^
Dietary fiber (g/d)	0.32^*^	0.33^*^	0.33^*^
Vitamin C (mg/d)	0.21^*^	0.22^*^	0.22^*^
Calcium m(g/d)	0.46^**^	0.51^**^	0.51^**^
Iron (g/d)	0.40^*^	0^*^.41	0.41^*^

De-attenuated, correlation coefficients adjusted for within-person variation using ANOVA to estimate within- and between-person variance and ratio

*p < 0.05, significant Spearman correlation

**p < 0.001, significant Spearman correlation

The main reason given for not wishing to participate in this validation study (54 out of 178 invited) was lack of time. When we examined characteristics of participants (*n* = 124) and non-participants (*n* = 54), no substantial differences were found for sex, age, BMI, proportion born to GDM mothers, or parental education. Within our set of participants, we observed no significant differences in dietary intakes between offspring born to GDM and non-GDM mothers for all dietary variables tested except for vegetables (mean difference of: 25 g/d, highest among offspring born to GDM mothers) (data not shown). Overall, minor differences in dietary habits between overweight- and normal-weight participants were found: the mean difference between the FFQ and the 3x24HRs was significantly higher for energy from protein (1.3E%) and significantly lower for carbohydrate (−3.7E%) among overweight children compared with normal weight children (Additional file 1: Table S1). Furthermore, comparing our study population in which GDMmothers are overrepresented (and not participating in the DNBC follow-up, *n* = 76) with the adolescents from the DNBC 14-year follow-up (*n* = 24,879) we found no significant difference for proportion of girls (47% vs. 52%, *p* = 0.10), parental education (*p* = 0.11), and maternal smoking (*p* = 0.13). However, children in the validation study were significantly younger (7 months, *p* < 0.0001), and were more frequently overweight/obese (16% vs. 9%, *p* = 0.03) compared with the adolescents from the DNBC 14-year follow-up. No significant differences in nutrient intake on a group level were observed across the three 24HRs (data not shown).

## Discussion

Comparing dietary intake assessed by the web-based FFQ with that of three 24HRs among Danish adolescents we found that four (fish, fruits, vegetables, and oils and dressing) out of 10 food groups and three (protein E%, dietary fibres, and vitamin C) of the 11 nutrients were overestimated by the FFQ. This is similar to what has often been reported in similar studies in adolescents [13, 33, 34]. Total energy intake did not differ significantly between the two methods which is comparable to what was found in the validation study of the original GUTS questionnaire [13]. However, in the validation study by Rockett et al. mean Pearson correlation coefficients for nutrients were 0.41 compared with 0.35 in our study (Spearman’s correlation coefficients). Even though the agreement estimated by the kappa statistics primarily was poor to fair/moderate (ranging from 0.05–0.43 [35]), the ability to rank adolescents according to magnitude of intake was good in the sense that misclassification (in the opposite quartile) below 10% was seen for most food groups and nutrients, except for fish and carbohydrate E% [31]. Misclassification below 5% was seen for oils and dressing, fatty acids, and iron.

When comparing our results with a validation study performed using a similar FFQ to ours in the Norwegian mother-child cohort, we found a lower level of misclassification into opposite quartile in general. However, it must be noted that the Norwegian study used quintiles [12].

Intake of fish, fruits, and sweets differed significantly between the two methods, with higher intake of fruits and fish and lower intake of sweets in the FFQ relative to the 24HRs. Fish is often rarely consumed and may not be captured by only 3 days of dietary recording, as was also observed in other studies [12]. Even though the three randomly assigned 24HRs covered all 7 weekdays, three 24HRs might not have sufficiently estimated the day-to-day variability [9] of e.g. fish, resulting in large median difference and low correlation between the two methods. Further, a high number of subjects were misclassified in the opposite quartile of intake which was also observed in the study by Overby et al. [12]. According to a Danish study using dietary data from The Danish National Survey of Dietary Habits and Physical Activity 2003–2008, intake of sweets, cakes, and sugar-sweetened beverages among 789 children aged 4–14 years were higher during weekends compared with weekdays [36]. Together with the fact that adolescents were found to be more prone to omit foods compared with adults [19], the lower estimated intake of sweets in the FFQ might be a result of intake during weekends being omitted by the adolescents when completing the FFQ.

The percentage difference in intake between the FFQ and 3x24HRs of 121% for fruits (highest in the FFQ) and a correlation coefficient of 0.13 for vegetables deserves some attention. A relatively large intervention study among 9-10-year-old Danish children (*n* = 798) reported a median (p10, p90) intake of fruits of 126 g/d (38, 244) and a median (p10, p90) intake of vegetables of 126 g/d (54, 227) measured by 7d food records [37]. The level of intake is comparable to the median (p25, p75) intake of fruits estimated with our FFQ; 126 g/d (52–247). Additionally, the Danish study showed a significantly higher consumption during school hours compared with outside school [37]. Further, a study among 96 adolescents aged 11 to 15 years found only 30% match between a 24HR method and direct observations [38]. Therefore, it could be speculated that meals consumed during school hours were omitted during 24HRs and fruits and vegetables were underestimated by the 24HRs. However, since prompt questions were included in the 24HRs to ensure that intake during school hours was captured, intake of fruits may have been overestimated by the FFQ as a consequence of a high number of questions about fruits in the FFQ. Variation in season should, furthermore, also be considered. The FFQ was completed from December to March whereas the 24HRs were conducted between January and June. Thus, there were some overlapping periods at group level. If season had had a substantial impact, we would have expected higher intakes of fruits and vegetables estimated from the 24HRs in the spring. However, median intakes of fruits and vegetables were lower in the 24HRs compared with the FFQ.

The observed disparities in mean intakes of food groups and low percentages classified into same quartile were to some extent expected due to the adolescent study population. Adolescents are known to pay less attention to dietary habits, and their snacking and eating habits tend to be more unstructured compared with those of adults. They may also be less motivated to record their dietary intake and have increased focus on body image, which seems to affect the accuracy of their self-report [39]. These characteristics may result in high within-person day-to-day variability among adolescents which seems to attenuate correlation coefficients [9, 14]. This was in particular evident for dairy, energy intake, MUFA, and calcium where Spearman’s correlation coefficient increased after de-attenuation which was also reported in the study by Rockett et al. [13]. However, Spearman’s correlation coefficients for bread, fish and carbohydrate E% did not improve after de-attenuation. This suggests that intake of bread and fish was reported consistently over time, whereas other intakes varied more among the participating adolescents. De-attenuation improved an already strong correlation coefficient for dairy, indicating some variation between days nevertheless; recall of milk products was rather constant. Studies have reported that adolescents might have some difficulties in combining amount of foods eaten and frequency [18] which could also have contributed to the observed level of inconsistencies across foods and nutrients.

Energy adjustment did not affect level of correlation coefficients substantially in any direction, indicating that intake was not related to systematic errors of under- or over estimation of intake, nor was nutrient intake related to energy intake [9]. Difficulties in reaching strong correlation coefficients among adolescents were presented in a systematic review where only 2 of the 18 validity studies reported mean correlation coefficients above 0.50 [40]. Recall periods of 1 day or 1 week and fewer than 70 food items in the FFQ were assigned as main reasons for stronger correlation coefficients [40]. However, in a recent meta-analysis by Tabacchi et al., correlation coefficients for most nutrients in 16 validity studies were above 0.40, and it was suggested that the number of food items should not exceed 114 [10]. We cannot rule out that the number of food items in our FFQ (145) could be a contributor to the observed overestimation of some food groups. However, the correlation coefficient of e.g. fruits was 0.40. Nonetheless, both similar and stronger correlation coefficients ranging from ~0 to 0.8 have been reported for nutrients and food groups in other validation studies among adolescents [12, 14, 34]. Also in adults, higher correlation coefficients have been observed [41, 42] which could be due to better skills in frequency estimation, better memory of consumed foods, and better knowledge to identify foods compared with those of children [18]. This was to some extent confirmed in a study among 11- to 15-year-old children accordingly to which unfamiliar food terms, unfamiliar measurements, and poor knowledge of food fractions made recording diet problematic using a web-based 24HR [38]. Training of recalling diet among children has previously been suggested by Lu and colleagues in order to improve accuracy [43]. It could therefore be speculated whether training in recalling diet before the actual dietary assessment with an FFQ could increase both motivation and food knowledge among the adolescent population and thereby improve the quality of the results.

This validation study has several strengths. The web-based FFQ was easy to administer and it may increase participant motivation that recording takes less than 1 h, thereby potentially increasing participation rate [44]. The time interval of 4 weeks between the FFQ and the 24HRs seemed adequate to avoid carryover effects from the FFQ to the 24HRs [45]. Three non-consecutive 24HRs were chosen as reference method, because these have been suggested to be sufficient to assess habitual energy intake [46]. Since adolescents seem to have high within-person variation in diet, de-attenuated correlation coefficients were calculated in order to obtain an estimate similar to that gained with a higher number of 24HRs [9]. We ensured high quality of the 24HRs with four trained project team members conducting the interviews. Recalls by telephone have shown to yield results similar to those of face-to-face interviews [47]. Moreover, this method was less expensive and we were able to reach participants from across the country [48]. Furthermore, telephone interviews potentially decreased the correlation between the test and reference method [49]. With the allocation of interview days, we secured representation of all weekdays at group level. Nevertheless, due to adolescents’ eating habits, some food items might not have been captured sufficiently. When we report correlation coefficients between the FFQ and the three 24HRs that rely on individual responses to these assessments, the number of days are not equally distributed between study participants. Since the days were randomly allocated this should not bias our estimates.

Limitations of the present study are firstly the different recall periods of the FFQ and the 24HRs. This could potentially increase the differences in observed estimates, as the FFQ was a self-administered long-term recall compared with the interview-based prompted short-term 24HR. Secondly, the 12–15-year age range of both boys and girls may have contributed to the relatively large variability within the study population and thus overall have lowered the level of relative validity. Thirdly, the FFQ and 24HR methods share measurement errors, e.g. both methods rely on memory [50]. Therefore, more objective measures, though also subject to measurement errors, such as observations in school or the double labelled water method, could have added an objective validation perspective of the FFQ tested. Finally, the fact that cohorts are often oversampled with a higher level of socio-economic status, and that 62% of the present study population were born to GDMmothers may render the results of the present study less representative for the Danish adolescent population with respect to e.g. proportion of overweight/obese adolescents [51, 52]. However, comparisons between our participants and the entire DNBC cohort of 14-year-olds (currently participated *n* = 24,879) revealed relatively minor differences. In these analyses, the difference in age was relatively small and is due to the recruitment of children younger than 14 years of age, which was necessary to increase sample size in the validation study.

## Conclusion

The relative validity of the FFQ compared with the 3x24HRs showed that the ranking ability differed across food groups and nutrients. When considering the classification in quartiles and the Spearman’s correlation coefficients together, the relative validity showed that the ranking ability was acceptable for estimated intake of dairy, fruits, oils and dressing, SFA, MUFA and iron. No major misclassification was observed for oils and dressings and fatty acids. Larger variation was seen for fish, sweets, and vegetables, and caution should be taken when interpreting estimation from the FFQ of fish and carbohydrate E%. When evaluating estimates from the web-based FFQ among Danish adolescents these findings should be considered.

## Additional file

Additional file 1: Table S1. Mean intake of food groups, energy and nutrients from FFQ and 3x24HR stratified by BMI categories. (DOCX 14 kb)

## Abbreviations

24HRs: 24-h recalls
BMI: Body Mass Index
DNBC: Danish national birth cohort
E%: Energy percent
FFQ: Food frequency questionnaire
GDM: Gestational diabetes mellitus
GUTS: Growing up today study
GW: Gestation week
IOTF: International obesity task force
MUFA: Mono-unsaturated fatty acids
NHS II: Nurses’ health study II
PUFA: Polyunsaturated fatty acids
SD: Standard deviation
SFA: Saturated fatty acids
YAQ: Youth/adolescent questionnaire
