# Supplementary material for: Relative validity of a web-based food frequency questionnaire for Danish adolescents
Source: Nutr J. 2018 Jan 12;17:9. doi: 10.1186/s12937-018-0312-7 (PMC5767066; doi:10.1186/s12937-018-0312-7)
Supplement: Additional file 1: Table S1. — Mean intake of food groups, energy and nutrients from FFQ and 3x24HR stratified by BMI categories. (DOCX 14 kb) [file 12937_2018_312_MOESM1_ESM.docx]

## Additional file 1: Table S1 Mean intake of food groups, energy and nutrients from FFQ and 3x24HR stratified by BMI categories

|  | BMI <25 (n=90) | | BMI >25 (n=34) | |
| --- | --- | --- | --- | --- |
|  | Mean | | Mean | |
| Food groups (g/d) | FFQ | 24HR | FFQ | 24HR |
| Beverages | 1030 | 1139 | 1107 | 1232 |
| Dairy | 412 | 373 | 532 | 365 |
| Bread | 286 | 214 | 321 | 202 |
| Cereals | 36 | 37 | 28 | 26 |
| Meats/poultry | 91 | 113 | 100 | 99 |
| Fish | 23 | 11 | 11 | 13 |
| Fruit | 216 | 97 | 164 | 78 |
| Vegetable | 144 | 113 | 134 | 89 |
| Sweets | 24 | 83 | 19 | 68 |
| Oils & dressing | 39 | 35 | 37 | 32 |
|  |  |  |  |  |
| Nutrients |  |  |  |  |
| Energy (MJ/d) | 9.2 | 9.4 | 9.0 | 8.4 |
| Protein (E%)^b^ | 14^a^ | 13 | 16^a^ | 13 |
| Fat (E%) | 33 | 35^a^ | 33 | 33^a^ |
| SFA (g/d) | 29 | 36^a^ | 27 | 29^a^ |
| MUFA (g/d)^b^ | 26 | 30 | 26 | 25 |
| PUFA (g/d) | 14 | 12 | 13 | 12 |
| Carbohydrate (E%)^b^ | 51 | 50^a^ | 50 | 53^a^ |
| Added sugar (g/d) | 34 | 50 | 28 | 48 |
| Dietary fiber (g/d) | 29 | 20 | 28 | 19 |
| Vitamin C (mg/d) | 88 | 54 | 77 | 50 |
| Calcium (mg/d)^b^ | 998 | 950 | 1143 | 877 |
| Iron (mg/d) | 10 | 9 | 10 | 8 |

^a^ Intake with the FFQ *or* 3x24HR was significantly different between BMI strata

^b^ Mean difference between FFQ and 3x24HR was significantly different between BMI strata

Differences were tested using Kruskal-Wallis test on skewed data (all variables) except macro-nutrients

where ANOVA was applied

FFQ, food frequency questionnaire; 24HR, 24-hour recalls; SFA, saturated fatty acids;

MUFA, monounsaturated fatty acids; PUFA, polyunsaturated fatty acids
